# Control of anthocyanin and non-flavonoid compounds by anthocyanin-regulating MYB and bHLH transcription factors in *Nicotiana benthamiana* leaves

**DOI:** 10.3389/fpls.2014.00519

**Published:** 2014-10-08

**Authors:** Nikolay S. Outchkourov, Carlos A. Carollo, Victoria Gomez-Roldan, Ric C. H. de Vos, Dirk Bosch, Robert D. Hall, Jules Beekwilder

**Affiliations:** ^1^Business Unit Bioscience, Plant Research International, Wageningen University and Research CentreWageningen, Netherlands; ^2^Laboratory of Plant Physiology, Wageningen University and Research CentreWageningen, Netherlands; ^3^Laboratory of Pharmacognosy, Federal University of Mato Grosso do Sul, Campo GrandeBrazil

**Keywords:** anthocyanin, MYB, bHLH, polyamines, octanoyl-nornicotine, *N. benthamiana*

## Abstract

Coloration of plant organs such as fruit, leaves and flowers through anthocyanin production is governed by a combination of MYB and bHLH type transcription factors (TFs). In this study we introduced Rosea1 (ROS1, a MYB type) and Delila (DEL, a bHLH type), into *Nicotiana benthamiana* leaves by agroinfiltration. ROS1 and DEL form a pair of well-characterized TFs from Snapdragon (*Antirrhinum majus*), which specifically induce anthocyanin accumulation when expressed in tomato fruit. In *N. benthamiana*, robust induction of a single anthocyanin, delphinidin-3-rutinoside (D3R) was observed after expression of both ROS1 and DEL. Surprisingly in addition to D3R, a range of additional metabolites were also strongly and specifically up-regulated upon expression of ROS1 and DEL. Except for the D3R, these induced compounds were not derived from the flavonoid pathway. Most notable among these are nornicotine conjugates with butanoyl, hexanoyl, and octanoyl hydrophobic moieties, and phenylpropanoid-polyamine conjugates such as caffeoyl putrescine. The defensive properties of the induced molecules were addressed in bioassays using the tobacco specialist lepidopteran insect *Manduca sexta*. Our study showed that the effect of ROS1 and DEL expression in *N. benthamiana* leaves extends beyond the flavonoid pathway. Apparently the same transcription factor may regulate different secondary metabolite pathways in different plant species.

## INTRODUCTION

Higher plants produce a large variety of low-molecular weight secondary compounds, such as phenylpropanoids, terpenoids, and alkaloids (Pichersky and Gang, 2000). Anthocyanins are colorants, that may appear red, purple, or blue depending on their chemical composition and the pH. Anthocyanins are a specific class of flavonoids synthesized via the phenylpropanoid pathway that have been part of the human diet in the form of fruit and berries since ancient times. There is increasing scientific evidence that anthocyanins provide health-promoting benefits (He and Giusti, 2010; Martin et al., 2011).

The patterns of anthocyanin coloration in different plant organs and tissues of higher plants is under the control of specific transcription factors (TFs) of the MYB and basic helix-loop-helix (bHLH) families (Petroni and Tonelli, 2011). MYB TFs that regulate anthocyanin biosynthesis belong to the R2R3-type MYB family (Stracke et al., 2001; Feller et al., 2011). Members of this protein family regulate diverse processes such as the phenylpropanoid pathway, tryptophan biosynthesis, epithelial cell fate identity and plant responses to environmental factors, and they also play a role in mediating hormone actions (Stracke et al., 2001; Broun, 2005; Dubos et al., 2010). R2R3-MYB proteins execute their regulatory functions through physical association with bHLH types of TFs and a WD repeat protein (Ramsay and Glover, 2005). Upon ectopic expression, many of the R2R3-MYB TFs, e.g., Rosea1 (ROS1) from *Antirrhinum majus* (Schwinn et al., 2006), *Arabidopsis* MYB75/PAP1 (Kranz et al., 1998) or the maize C1 (Cone et al., 1986), are able to induce anthocyanin production in a variety of divergent mono- and dicot plant species such as tomato, *Arabidopsis*, petunia and maize (Cone et al., 1986; Spelt et al., 2000; Teng et al., 2005; Butelli et al., 2008). For the activity of ROS1, a bHLH factor, such as Delila (DEL) from *A. majus*, is often required (Butelli et al., 2008) although in tobacco it has been shown that the expression of ROS1 alone could mediate anthocyanin formation (Orzaez et al., 2009).

There are many examples of TFs that execute the same function in different plant species. It has been described that MYB TFs such as ROS1 and PAP1 can induce accumulation of specific anthocyanins (Tohge et al., 2005; Butelli et al., 2008; Bhargava et al., 2013). Knowledge of the effects of such TFs on metabolites from different pathways has been scarce. It has been described that the overexpression of PAP1 in *Arabidopsis* leads to accumulation of anthocyanins, and concomitantly to a decrease in secondary cell wall components such as lignin and polysaccharides (Bhargava et al., 2013), and also of procyanidins (Tohge et al., 2005). On the other hand, compounds more closely related to anthocyanins, such as flavonols, have been described to be co-induced with anthocyanins (Butelli et al., 2008). *Nicotiana* is a plant genus that is known for its broad set of defensive molecules, including phenolic (Gaquerel et al., 2014) and nicotine-derived compounds (Dawson, 1945), and for its ability to produce anthocyanins (Deluc et al., 2006). It is therefore an interesting platform to monitor cross-talk between different metabolite groups upon overexpression of TFs regulating secondary metabolism.

In this work, the effects of expression of ROS1 and DEL from *A. majus* on *Nicotiana benthamiana* metabolites was estimated using untargeted LC-MS analysis. In line with their well-characterized role as anthocyanin-specific TFs, ROS1 and DEL overexpression resulted in the accumulation of the anthocyanin delphinidin 3-rutinoside (D3R). Surprisingly, accumulation of phenolamides as well as nornicotine conjugates with butan-, hexan-, and octan-oyl hydrophobic moieties was also observed. These compounds are defence *N. benthamiana* compounds with an established activity against insect herbivores (Severson et al., 1988; Kaur et al., 2010). Our study has shown that the effect of ROS1 and DEL expression in *N. benthamiana* therefore extends beyond anthocyanin production. Apparently, the same transcription factor pair can activate different secondary metabolite pathways in different plant species.

## MATERIALS AND METHODS

### MATERIALS AND CONSTRUCTS

Standards of delphindin 3-rutinoside (Extrasynthese, Genay, France), chlorogenic acid, nicotine, and tryptophan (Sigma, St Louis, MO, USA) were used in concentrations ranging from 3.12 to 200 μg/ml. cDNAs from ROS1 and DEL were amplified by PCR using genomic DNA of E8:ROS/DEL tomato fruits obtained from (Butelli et al., 2008). PCR products were gel purified and TOPO cloned into the pCR8/GW/TOPO-TA vector (Invitrogen). After sequence verification the ROS1 and DEL fragments were transferred by GATEWAY recombination to pK7WG2 ^1^ to create 35S-ROS1 and 35S-DEL. The plasmids obtained were then introduced into *Agrobacterium tumefaciens* AGL0 (Lazo et al., 1991). AGLO harboring pBINPLUS (pBIN) plasmid (van Engelen et al., 1995) was used as a negative control.

### AGROINFILTRATION

*Nicotiana benthamiana* infiltrations were carried out as described previously (van Herpen et al., 2010). Briefly, *Agrobacterium* strains were grown at 28°C for 24 h in LB medium with antibiotics. Cells were resuspended in MES buffer (10 mM MES; 10 mM MgCl2; 100 mM acetosyringone) to a final OD_600_ of 0.5, followed by incubation for 150 min. *Agrobacterium* strains (pBIN, 35S-ROS1 and/or 35S-DEL) were mixed in equal volumes. *N. benthamiana* plants were grown in a greenhouse with 16 h light at 28°C. Strain mixtures were infiltrated into the abaxial side of leaves of four-week-old plants using a 1 mL syringe. The plants were grown under greenhouse conditions before further analysis.

For each reported metabolite, data derive from three independent infiltration experiments, performed on different dates. Within each infiltration experiment, the same *Agrobacterium* solutions were used for three independent leaves on different *N. benthamiana* plants. Each leaf was analyzed individually.

### METABOLITE ANALYSIS

Metabolite analysis was essentially performed as described in De Vos et al. (2007). Leaves were harvested 5 days post infiltration, snapfrozen in liquid nitrogen and ground to a fine powder. Exactly 200 mg (+/–2 mg) of powder was extracted with 800 μL of methanol containing 1% formic acid. Extracts were sonicated for 15 min, centrifuged at 12500×*g* for 10 min and filtered through 0.45 μm filters (Minisart SRP4, Biotech GmbH, Germany). An Accela High Pressure Liquid Chromatography system with a photodiode array (HPLC-PDA; Thermo) coupled to an LTQ Ion Trap-Orbitrap Fourier Transformed Mass Spectrometer (FTMS; Thermo) hybrid system was used to detect, identify and quantify compounds (van der Hooft et al., 2012a). A LUNA 3 μ C18 (2) 150 × 2.00 mm column (Phenomenex, USA) was used to separate the extracted metabolites, with MQ water with 0.1% formic acid (A) and acetonitrile with 0.1% formic acid (B) as solvents. A linear gradient from 5 to 35% B at a flow rate of 0.19 ml/min was used. The FTMS was set at a mass resolution of 60,000 HWHM and a mass range of m/z 85-1200, using electrospray ionization in positive mode. Identification of detected compounds was based on retention time, accurate masses of both the parent and fragment ions, in combination with any PDA absorbance spectra (recorded at 240–600 nm).

Data analysis was performed in an untargeted manner, essentially as described (De Vos et al., 2007). Visualization of the HPLC-PDA-FTMS data was performed using Xcalibur 2.1 software (Thermo). The MetAlign software package ^2^ was used for baseline correction, noise estimation, and mass peak alignment (Lommen, 2009). Threshold for peak detection was set at 10000 ions per scan. Mass peaks originating from the same metabolite, including adducts, fragments and isotopes, were subsequently clustered into so-called reconstructed metabolites, using MSClust software (Tikunov et al., 2012). Zero values for compound intensity, i.e., absent in sample, were replaced by random values between 0 and 1. The resulting relative intensity levels of compounds were then used for further statistical analysis, using Student’s *t*-test. Metabolites of interest were annotated by querying literature data for metabolites, previously detected in *N. benthamiana* or other *Nicotiana* species. In addition, we checked for the presence of compounds that have previously been identified from other plant sources and analyzed at the same LC-MS conditions (Moco et al., 2007; van der Hooft et al., 2012b). Quantification of selected compounds in the leaf extracts was performed using the same HPLC-PDA-Orbitrap FTMS system and conditions, using dilution series of authentic standards.

### INSECT ASSAYS

*Manduca sexta* bioassays were carried out as follows. Infiltrated *N. benthamiana* leaves were collected after 4 days of infiltration. Leaves were placed in Petri dishes with a wet filter paper and their petioles were placed in Eppendorf tubes that contained 1% agar in water. Per infiltration construct four replicates were used, and every replicate included five larvae. Leaves and larvae were incubated in the dark at 25°C. Leaves were replaced on day 3, and on day 5 the weights of individual larvae were recorded.

## RESULTS

### PHENOLIC AND NON-PHENOLIC MOLECULES REGULATED BY ROS1 AND DEL IN *N. benthamiana*

**FIGURE 1:**
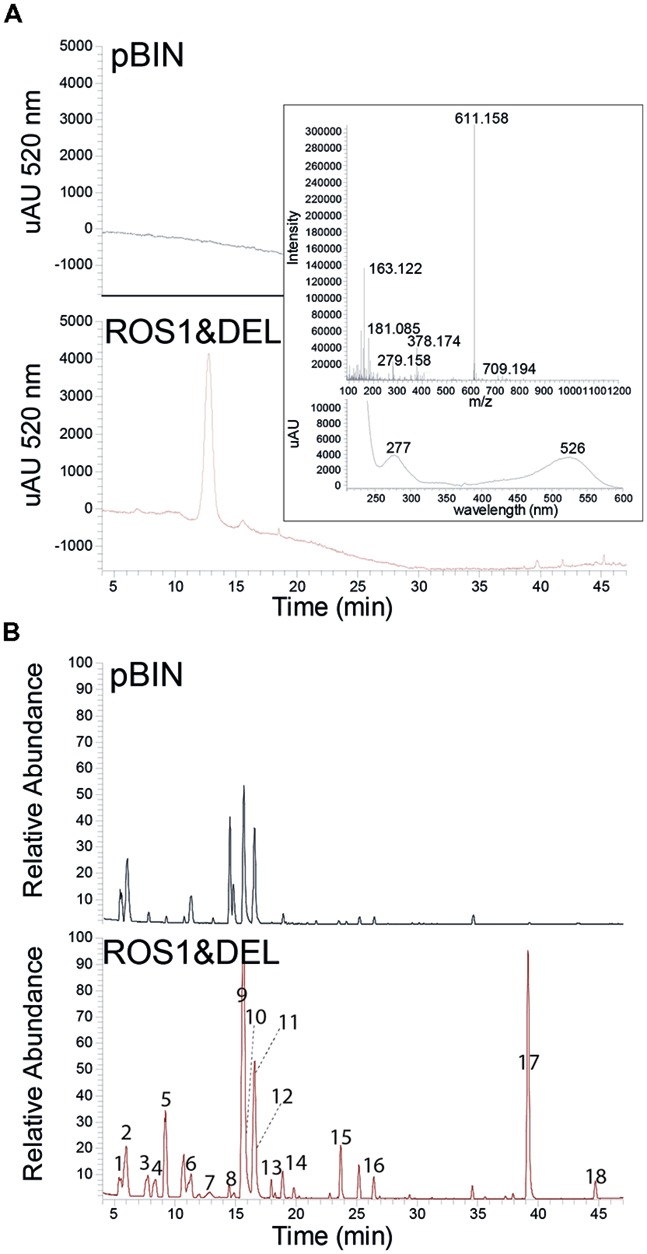
**(A)** HPLC-Photodiode array detection at 520 nm of extracts from pBIN (control) and ROS1&DEL infiltrated *Nicotiana benthamiana* leaves. Inserted panel shows the detected accurate mass and the UV-Vis absorbance spectrum of the delphinidin-3-rutinoside (D3R) peak. **(B)** HPLC-MS chromatograms of the samples described in **(A)**. The relative abundance of mass signals is, in both cases, 1e7 at 100%. Indicated peak numbers refer to **Table 1**.

**FIGURE 2:**
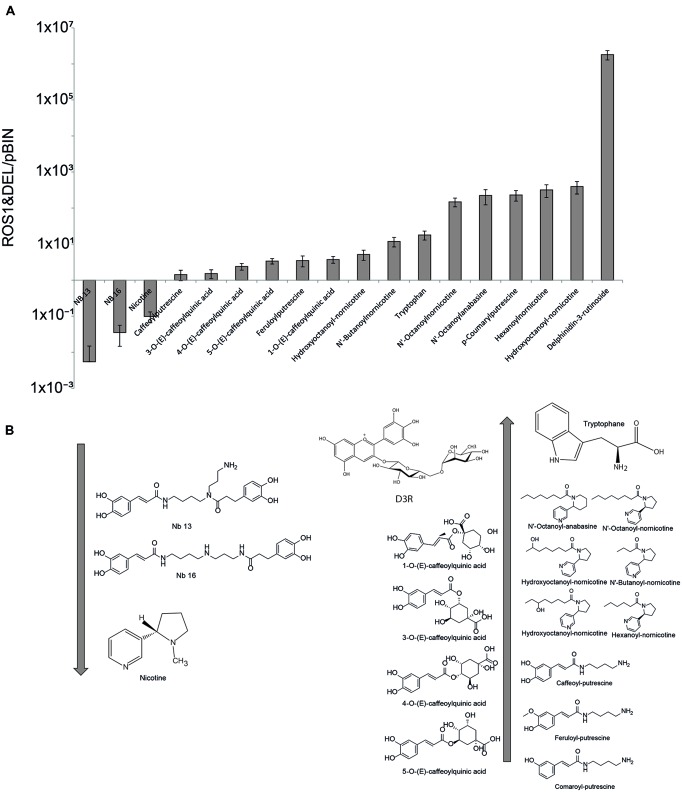
**(A)** Diagram showing the ratios of the MS signals of significantly changed (*p* < 0.05) compounds in the ROS1&DEL infiltrated leaves as compared to the empty vector pBIN. **(B)** Chemical structures of the down-regulated **(left)** and up-regulated **(right)** compounds.

We set out to investigate the effect of ROS1 and DEL in *N. bentamiana* on metabolite profiles by transient expression using agroinfiltration. Leaves of four-week-old *N. benthamiana* plants were infiltrated with *Agrobacterium* suspensions and harvested, unless indicated, 5 days after infiltration. Anthocyanin formation could be observed by occurrence of a purple color in the leaves, but no other phenomena could be observed that would distinguish a ROS1 and DEL infiltrated leaf from a pBIN infiltrated leaf (supplemental Figure S1). Aqueous-methanol extracts of the leaves were subjected to HPLC-PDA-Orbitrap FTMS based global profiling, enabling the detection of a large variety of secondary metabolites, including anthocyanins (De Vos et al., 2007). Infiltration of ROS1 and DEL, induced a single peak with UV/Vis-absorbance at 520 nm, as detected by the photodiode array detector (PDA). This peak displayed a typical anthocyanin absorption spectrum and a monoisotopic mass of [M+H] = 611.158 m/z (**Figure 1A**, inserted panel). The observed m/z, retention time, absorption spectrum, and MSMS fragmentation pattern (**Table 1**) corresponded to that of the delphinidin 3-rutinoside (D3R) standard. Surprisingly, however, the LC-MS analysis revealed that, next to D3R, a range of other compounds not absorbing light at 520 nm, were also highly upregulated by ROS1 and DEL expression (**Figure 1B**). To investigate the reproducibility of induction of these compounds, we performed two new independent infiltration experiments, each in three replicates. Data were processed in an untargeted manner and analyzed for compounds that were significantly altered (students *t*-test, *p* < 0.05) in ROS1 and DEL infiltrated plants as compared to the control pBIN plants (**Table 1**). Most of the induced compounds were already present in the control pBIN plants, and could be annotated by comparison with authentic standards, or by MSMS analysis and comparison to literature data of *Nicotiana* compounds. **Figure 2** illustrates their relative changes in content induced by ROS1 and DEL infiltration.

**Table 1:** List of significantly changing compounds (students *t*-test, *p* < 0.05) in the ROS1&DEL infiltrated leaves as compared to pBIN (empty vector) infiltrated leaves.

	Putative identity	Ret. time (min)	[M+H]	λ max	Molecular formula	MSMS [M+H]	Identification	Reference
1	Nicotine	5.55	163.1230	nd	C_10_H_14_N_2_		Standard	
2	Caffeoylputrescine	6.06	251.1391	293	C_13_H_18_O_3_N_2_		Mass	Kaur et al. (2010)
3	Feruloylputrescine	7.83	265.1547	273	C_14_H_20_O_3_N_2_	89.11 [M-Feruloyl]; 177.05 [M-putrescin]	MSMS	Ben-Hayyim et al. (1994)
4	p-Coumarylputrescine	8.46	235.1442	293	C_13_H_18_O_2_N_2_	89.11 [M-coumaroyl]; 147.04 [M-putrescin]	MSMS	Gaquerel et al. (2013)
5	Tryptophan	9.3	205.0974	280	C_11_H_12_O_2_N_2_		Standard	
6	3-*O*-(E)-caffeoylquinic acid	11.39	355.1023	325	C_16_H_18_O_9_		Standard	
7	Delphinidin-3-rutinoside	12.86	611.1602	527	C_27_H_31_O_16_	465.10 [M-rhamnose]; 303.05 [M-rhamnose-glucose]	Standard, MSMS	Hugueney et al. (2009)
8	NB13*	14.88	472.2439	320	C25H_33_O_6_N_3_		Mass	Snook et al. (1988)
9	5-O-(E)-caffeoylquinic acid	15.69	355.1023	325	C_16_H_18_O_9_		Standard	
10	*N*′-Butanoylnornicotine	15.79	219.1493	298	C_13_H_18_ON_2_		Mass	Matsushita et al. (1979)
11	NB16**	16.42	472.2439	326	C_25_H_33_O_6_N_3_		Mass	Snook et al. (1988)
12	4-*O*-(E)-caffeoylquinic acid	16.57	355.1023	326	C_16_H_18_O_9_			
13	Hydroxyoctanoyl-nornicotine I	18.22	291.2066	293	C_17_H_26_O_2_N_2_		Mass	Miyano et al. (1981)
14	1-*O*-(E)-caffeoylquinic acid	18.89	355.1023	312	C_16_H_18_O_9_			
15	Hexanoylnornicotine	23.72	247.1805	262	C_15_H_22_ON_2_	229.17 [M-H_2_O]; 149.11 [M-hexanoyl]	MSMS	Bolt (1972)
16	Hydroxyoctanoyl-nornicotine II	26.57	291.2066	258	C_17_H_26_O_2_N_2_		Mass	Miyano et al. (1981)
17	*N*′-Octanoylnornicotine	39.26	275.2117	262	C_17_H_26_ON_2_	257.20 [M-H_2_O], 149.11 [M-octanoyl]	MSMS	Osawa et al. (1990)
18	*N*′-Octanoylanabasine	44.57	289.2273	270	C_18_H_28_ON_2_		Mass	Snook et al. (1988)

*NB13, *N*-(3-aminopropyl)-*N*-[4-[[(2E)-3-(3,4-dihydroxyphenyl)-1-oxo-2-propen-1-yl]amino]butyl]-3,4-dihydroxybenzenepropanamide; **NB16, *N*-[3-[[4-[[3-(3,4-dihydroxy phenyl)-1-oxo-2-propen-1-yl]amino]butyl]amino]propyl]-3,4-dihydroxybenzenepropanamide.

As expected, D3R was highly upregulated in the ROS1 and DEL infiltrated plants. In addition, a number of compounds which are derived from the phenylpropanoid pathway but which do not qualify as anthocyanins or flavonoids were detected. Among these was a group of phenolamides, consisting of hydroxycinnamic acids conjugated to polyamines such as p-coumaroyl-, caffeoyl-, and feruloyl-putrescine. On the other hand, down-regulation of two higher molecular weight caffeoyl-polyamine conjugates, i.e., N-(3-aminopropyl)-*N*-[4-[[(2E)-3-(3,4-dihydroxyphenyl)-1-oxo-2-propen-1-yl]amino]butyl]-3,4-dihydroxybenzenepropan amide, abbreviated as NB13, and *N*-[3-[[4-[[3-(3,4-dihydroxyphenyl)-1-oxo-2-propen-1-yl]amino]butyl]amino]propyl]-3,4-dihydroxybenzenepropanamide, abbreviated as NB16, were down-regulated (**Table 1**). Caffeoylquinic acids and tryptophan were found to be upregulated, indicating a general up-regulation of products of the shikimate pathway. Surprisingly, while nicotine levels were found to be down-regulated, a number of nornicotine conjugates carrying butanoyl, hexanoyl, and octanoyl hydrophobic moieties, which are not related to the phenylpropanoid pathway, were significantly (students *t*-test, *p* < 0.05) up-regulated.

Thus, ROS1 and DEL expression results in a range of both phenolic and non-phenolic metabolites in *N. benthamiana* (**Figure 2**). The mechanism of this induction (by direct gene activation of by indirect effects) remains elusive.

### ROS1 IS REQUIRED FOR THE INDUCTION OF NEW COMPOUNDS AND DEL ONLY POTENTIATES AND ENHANCES ROS1 ACTIVITY

**FIGURE 3:**
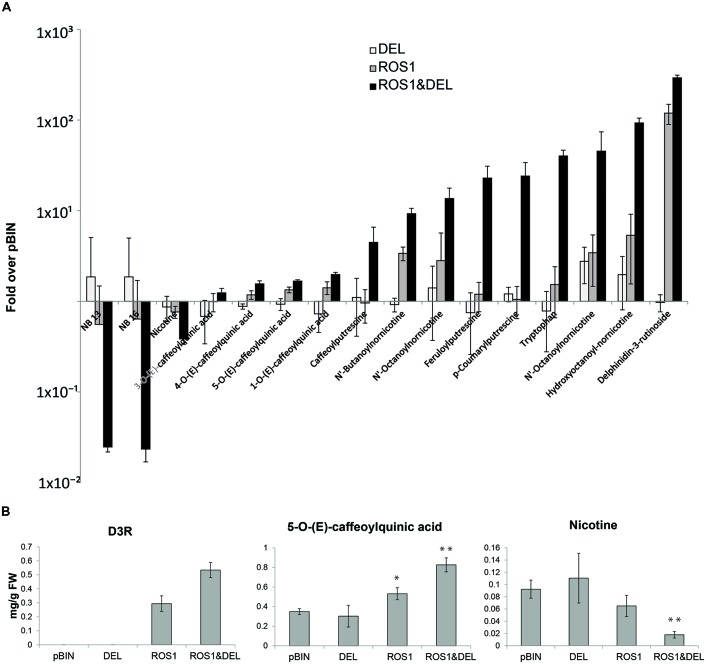
**(A)** Diagram showing the ratios of the MS signals of significantly changed compounds (students *t*-test, *p* < 0.05) in the leaves infiltrated with ROS1, DEL, and ROS1&DEL as compared to the empty vector pBIN. (**B)** Quantity, in milligrams/gram fresh weight (FW), of selected compounds present in **(A)** using a dilution series of commercially available standards. Stars indicate a significant change in the particular compound (Student’s *t*-test, *n* = 3; *p < 0.05; **p < 0.01) as compared to pBIN.

Earlier reports indicate that in *N. tabacum*, which is a close relative of *N. benthamiana*, ROS1 alone is sufficient for the induction of anthocyanins (Orzaez et al., 2009). We compared the effect of infiltration of combination of 35S-ROS1&35S-DEL against 35S-ROS1 alone and 35S-DEL alone on the global secondary metabolite profile (**Figure 3**). LC-MS analysis revealed that the anthocyanin D3R was still induced by ROS1, albeit two to four fold less than upon ROS&DEL co-infiltration. Other compounds, such as the putrescine conjugates and the nornicotine conjugates, were much less induced in the ROS1 only infiltration, while the DEL only infiltration hardly induced any changes in biochemical profile. In our interpretation, this indicated that ROS1 is essential for the changes in the metabolite profiles that were observed in *N. benthamiana*, while DEL is required for robust ROS1 activity. Levels of D3R, nicotine and 5-0-(E)-caffeoylquinic acid were quantified in the LC-MS using a dilution series of purified compounds (**Figure 3B**). D3R was undetectable in both the empty vector pBIN and DEL infiltrated plants. ROS1 infiltration alone yielded about 0.3 mg/g fresh weight of D3R. Combining ROS1 with DEL increased the D3R level about twofold. In control plants, the phenylpropanoid 5-0-(E)-caffeoylquinic acid was already present in well-detectable amounts. Infiltration of ROS1 alone increased its level significantly (students *t*-test, *p* < 0.05), while ROS&DEL co-infiltration showed doubled this effect, to 0.8 mg/g fresh weight. Thus, 5-0-(E)-caffeoyl quinic acid increased by a similar manner as D3R upon ROS1&DEL infiltration, relative to ROS1 alone. This result suggests that a general activation of the phenylpropanoid pathway has occurred, rather than a specific activation of the flavonoid/anthocyanin pathway. Nicotine levels were significantly (students *t*-test, *p* < 0.05) down-regulated by fivefold in the combination of ROS1&DEL, as compared to the control pBIN infiltrated leaves. Small nicotine down-regulation was also observed in ROS alone infiltration but that change was not significant.

### DEFENSIVE FUNCTIONS OF THE METABOLITES INDUCED BY ROS1 AND DEL

**FIGURE 4:**
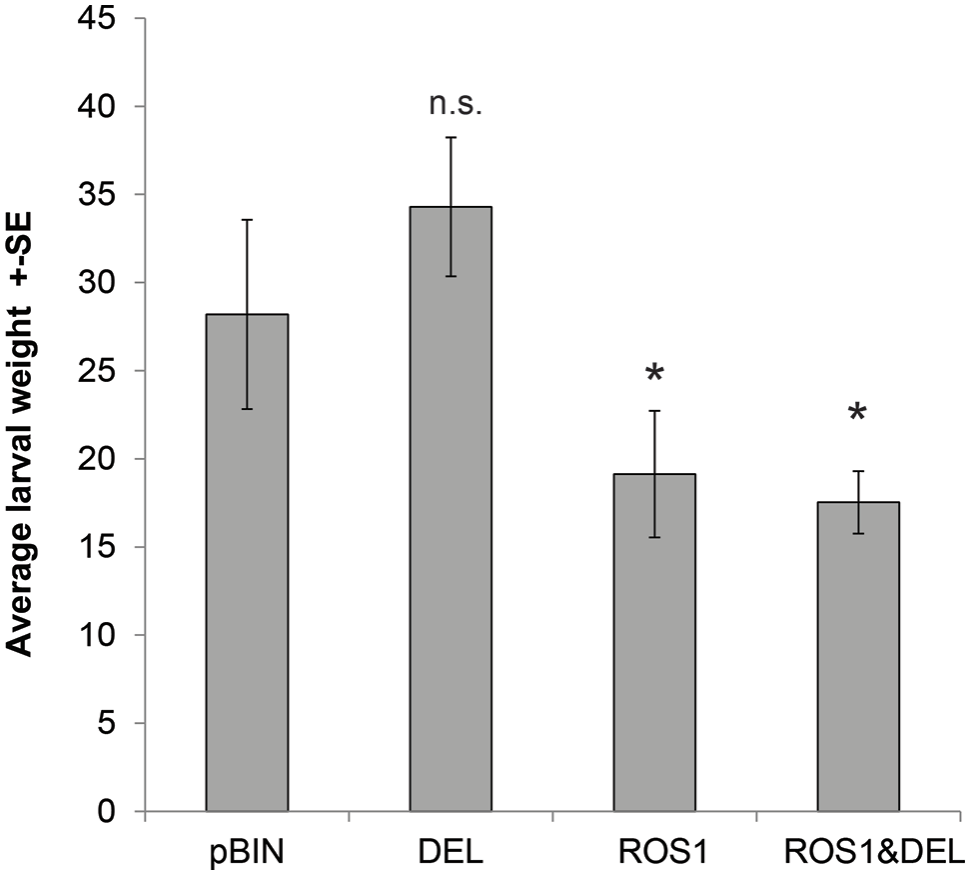
**Average larval weight of 20 larvae after 5 days of feeding on leaves infiltrated with the following constructs: pBIN, ROS1, DEL, and ROS1&DEL.** Star indicates a significant change (Student’s *t*-test, *n* = 20, *p* < 0.05) of the larval weight as compared to larvae fed on the empty vector pBIN treated leaves.

Both nornicotine conjugates and phenolamines have been described as plant defensive molecules against insect herbivores (Zador and Jones, 1986; Severson et al., 1988; Laue et al., 2000; Gaquerel et al., 2014). In addition there is a growing evidence that anthocyanin by itself might have a role in the plant defense against herbivores (Lev-Yadun and Gould, 2009; Markwick et al., 2013). We investigated if plant leaves ectopically expressing ROS1 and DEL exert altered resistance against the tobacco hornworm, *M. sexta*. *M. sexta* is a solanaceous specialist adapted to nicotine in tobacco, but not to phenolamines and octanoyl nornicotine (Severson et al., 1988; Kaur et al., 2010). We have fed *M. sexta* first instar larvae for 5 days with leaves infiltrated with ROS1 and DEL, both alone and in combination, and have compared them to pBIN controls. In **Figure 4**, the average larval weights after 5 days of feeding are shown. *M. sexta* larvae fed on pBIN or DEL infiltrated leaves did not differ significantly in weight. In contrast, a significant weight reduction (*t*-test, *p* < 0.05) was observed when larvae were fed on leaves expressing ROS1 or ROS1 and DEL.

These results indicate that anthocyanin-regulating ROS1 and DEL TFs act as defensive anti-herbivore TFs in *N. benthamiana.* The available data do not allow to distinguish the roles of the different compound classes in the anti-herbivore effect.

## DISCUSSION

In this study we report that ectopic expression of two anthocyanin-specific TFs from *A. majus* (ROS1 and DEL) in *N. benthamiana* induces, besides a single anthocyanin D3R, a range of non-flavonoid, defensive, anti-herbivore molecules. In contrast, in tomato and in *A. majus*, ROS1 and DEL appear to specifically induce anthocyanins and related flavonoids, while no other molecules of different chemical classes have been reported (Goodrich et al., 1992; Schwinn et al., 2006; Butelli et al., 2008). Most literature describing overexpression of related anthocyanin-regulating MYB TFs study only report changes in anthocyanin levels, while changes in other metabolites were often not studied (Aharoni et al., 2001; Kobayashi et al., 2002; Mathews et al., 2003; Vimolmangkang et al., 2013). *Arabidopsis* overexpressing MYB75/PAP1 showed reductions in cell-wall components (Bhargava et al., 2013), while an untargeted metabolome analysis showed predominant induction of anthocyanins and flavonols (Tohge et al., 2005).

The type of anthocyanins induced by ROS1 and DEL is much different in *N. benthamiana* leaves as compared to tomato fruit. In contrast to the single D3R in *N. benthamiana*, in tomato six different, glycosylated, methylated, and acylated forms of anthocyanin have been detected upon ROS1 and DEL expression (Butelli et al., 2008). This difference in anthocyanin types could be caused by a more limited anthocyanin modification potential in *N. benthamiana*, as compared to tomato. Indeed ectopic expression in *N. benthamiana* of MYB75/PAP1 (Hugueney et al., 2009) also resulted in D3R as the major anthocyanin accumulating in the leaves. Apparently, *N. benthamiana*, in contrast to tomato, does not display activity of anthocyanin methyltransferases, anthocyanin 5-glucosyltransferases and anthocyanin acyltransferases, required for specific anthocyanin modifications. Whether genes with these specific functions are absent from the *N. benthamiana* genome, or are not subject to regulation by these TFs is not clear.

While ROS1 is essential to induce accumulation of different compounds in *N. benthamiana*, our study suggests that DEL is only required to potentiate the functions of ROS1. It is likely that *N. benthamiana* produces an endogenous DEL-type of bHLH transcription factor, which can support activity of ROS1.

One of the major compound classes induced by ROS1 and DEL in *N. benthamiana* is the phenylpropanoid-polyamines. Studies in *N. attenuata* have revealed important functions of these compounds as plant defense factors protecting against insect herbivores (Gaquerel et al., 2014). Their biosynthesis is under control of NaMYB8 (Kaur et al., 2010). This TF is from the same R2R3-MYB family as ROS1, and seems to overlap to some extent in the secondary metabolism pathways it regulates. Similar to ROS1 in *N. benthamiana,* NaMYB8 in *N. attenuata* induces multiple phenylpropanoid-polyamine conjugates. While ROS1 induces formation of the anthocyanin D3R, NaMYB8 in *N. attenuata* induces formation of the flavonol quercetin rutinoside (Kaur et al., 2010). Interestingly, three hydroxycinnamoyl-coenzyme A: polyamine transferases from the BAHD family have been identified in *N. attenuata*, and they are strongly upregulated by NaMYB8 (Onkokesung et al., 2012).

Our study suggests a TF-regulated production of octanoyl-nornicotine and similar conjugates in plants. The insecticidal properties of these compounds (Severson et al., 1988) warrant an investigation into their biosynthesis and regulation. These molecules are not known to be regulated by NaMYB8 in *N. attenuata* (Kaur et al., 2010; Onkokesung et al., 2012). The biosynthesis of these nornicotine conjugates has been hypothesized to be directly linked to the pool of nicotine (Zador and Jones, 1986). Indeed we observed that increasing octanoyl-nornicotine and similar derivatives coincided with a reduction of the total unconjugated nicotine levels (). Nicotine is synthesized in the roots of multiple *Nicotiana* species. Transport to the aerial part of the plant is followed by its demethylation to nornicotine (Dawson, 1945). Subsequently, a yet unknown acyltransferase should transfer them to small fatty acid CoA conjugates. Acyl-nornicotine conjugates are normally present in the trichome exudate produced in the epidermis of the aerial parts of different *Nicotiana* species (Zador and Jones, 1986; Severson et al., 1988; Laue et al., 2000). Future experiments using ROS1 and DEL activation and gene expression studies may help to identify genes involved in the biosynthetic pathway toward acyl-nornicotine conjugates. It will be particularly interesting to identify the acyltransferase enzyme responsible for conjugation of nornicotine to acyl groups, and to compare its activity to both the *N. attenuata* acyltransferases involved in production of phenolic polyamine conjugates (Onkokesung et al., 2012) and putative tomato enzymes induced by ROS1 and DEL involved in anthocyanin acylation (Butelli et al., 2008).

In summary, the regulation of secondary metabolites by ROS1 and DEL in *N. benthamiana* extends beyond flavonoid production. ROS1 and DEL activate only partly understood pathways leading to the accumulation of phenolamines and nornicotine-conjugates. Therefore ROS1 and DEL have emerged as potentially valuable tools to investigate multiple secondary metabolite branches in different species.

## AUTHOR CONTRIBUTIONS

Nikolay S. Outchkourov designed de study, performed experiments, and wrote the manuscript. Carlos A. Carollo and Ric C. H. de Vos analyzed the LC-MS data. Victoria Gomez-Roldan generated the 35S-ROS1 and 35S-DEL constructs. Dirk Bosch, Ric C. H. de Vos, and Robert D. Hall facilitated the study and provided analysis and discussions. Jules Beekwilder provided discussions, data analysis, interpretations, and edited the manuscript.

## Conflict of Interest Statement

The authors declare that the research was conducted in the absence of any commercial or financial relationships that could be construed as a potential conflict of interest.

## Footnotes

1 http://gateway.psb.ugent.be

2 http://www.metAlign.nl

## SUPPLEMENTARY MATERIAL

Figure S1 **Picture of plants five days post infiltration.** On the left plant two leaves infiltrated with pBIN are indicated, serving as a control. On the right plant two leaves infiltrated with ROS1&DEL have been indicated. Note the darker coloration of the ROS1&DEL infiltrated leaves.
