# Supplementary figures and images for: Control of anthocyanin and non-flavonoid compounds by anthocyanin-regulating MYB and bHLH transcription factors in Nicotiana benthamiana leaves
Source: Front Plant Sci. 2014 Oct 8;5:519. doi: 10.3389/fpls.2014.00519 (PMC4189325; doi:10.3389/fpls.2014.00519)

Supplemental Figure S1

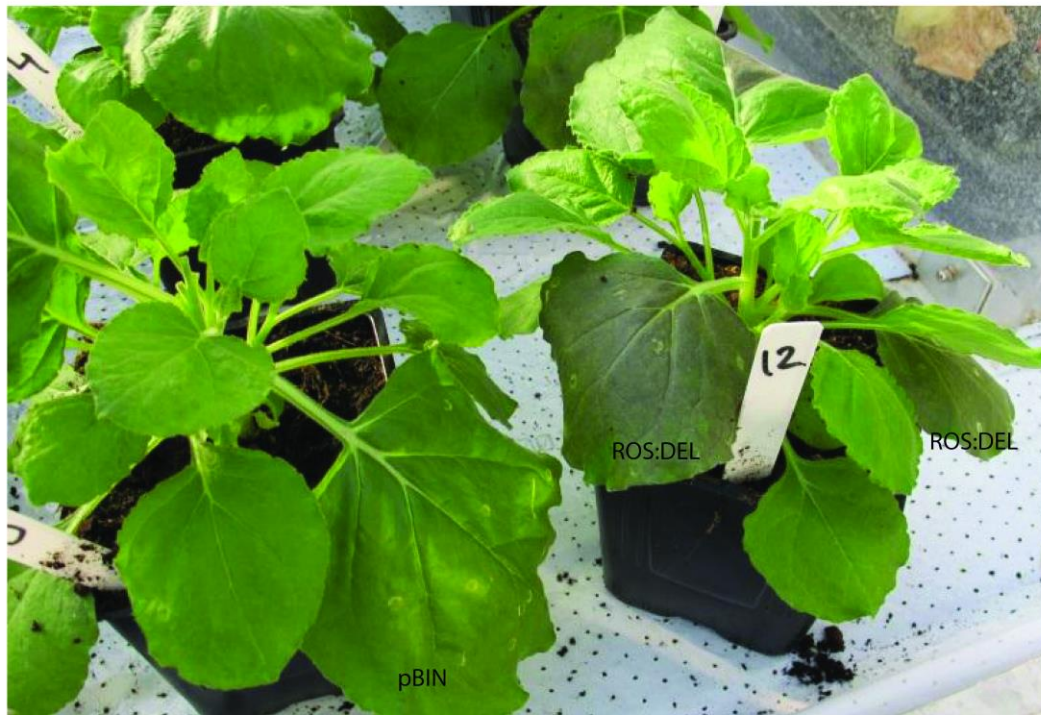

Supplement: Figure S1 — Picture of plants five days post infiltration. On the left plant two leaves infiltrated with pBIN are indicated, serving as a control. On the right plant two leaves infiltrated with ROS1&DEL have been indicated. Note the darker coloration of the ROS1&DEL infiltrated leaves. [file Presentation_1.PDF]
